# 

**Published:** 1880-05

**Authors:** 


MEETINGS OF DENTAL SOCIETIES IN JUNE:

California State Dental Association, 8th, at San Rafael.
Tennessee Dental Association, 30th, at Nashville.
Missouri Dental Association, 1st, at Sweet Springs.
Kentucky State Dental Association, 1st, at Louisville.
Indiana State Dental Society, 29th, at Franklin.
Massachusetts Dental Society, 3d, at 167 Tremont St., Boston.
Connecticut Valley Dental Society, 17th, at Savin Rock.

### HYMENEAL.

" Domestic happiness, thou only bliss
Of paradise that has survived the fall."

At Cranberry, N. J., March 23, 1880, by Rev. Joseph Van Dyke, E. H. Bergen, M. D., of Princeton, and Annie L., daughter of Ezekiel Slivus.

### NATALITIAL.

" Of all the joys that brighten suffering earth,
What joy is welcomed like a new-born child."

On Easter Monday last a son was born to Dr. and Mrs. E. F. Wayman, of Waterloo, Va.

### UNITED STATES POSTAL LAW.

Many persons who receive pamphlets, papers, etc., through the mails are unaware of the United States laws relating to them, and such will feel indebted to us for the information and guidance the enactments will afford which we copy below, viz.:

1. A postmaster is required to give notice *by letter* (returning a paper does not answer the law) when a subscriber does not take his paper out of the office, and state the reason for its not being taken. Any neglect to do so makes the postmaster *responsible* to the publishers for payment.

2. Any person who takes a paper from the post-office, whether directed to his name or another, or whether he has subscribed or not, is responsible for the pay.

3. If a person orders his paper discontinued, he must pay all arrearages, or the publisher may continue to send it until payment is made, and collect the whole amount, *whether it be taken from the office or not.* There can be no legal discontinuance until the payment is made.

4. If the subscriber orders his paper to be stopped at a certain time, and the publisher continues to send, the subscriber is bound to pay for it *if he takes it out of the post-office.* The law proceeds upon the ground that a man must pay for what he uses.

5. The courts have decided that refusing to take a newspaper and periodicals from the post-office, or removing and leaving them uncalled for, is *prima facie* evidence of intentional fraud.

This column will be sold for advertising space six months during the year at *special rates.*

N. B.—The Independent Practitioner will be received by a large number of persons for the *first* time, all of whom we trust will be induced to become subscribers. They will please remit NOW the amount of subscription ($2.00) to insure the continuation of the journal. Money can be sent at our risk by *post-office money order*, registered letter or check on banks in *N. Y. City* or *Baltimore*, *made payable to Dr. B. M. Wilkerson.* We hope that the few who have been receiving the journal and have not "paid up" will cheerfully do so *now.*

